# N-acetylcysteine Amide AD4/NACA and Thioredoxin Mimetic Peptides Inhibit Platelet Aggregation and Protect against Oxidative Stress

**DOI:** 10.3390/antiox12071395

**Published:** 2023-07-07

**Authors:** Sonia Eligini, Marco Munno, Daphne Atlas, Cristina Banfi

**Affiliations:** 1 Centro Cardiologico Monzino IRCCS, Unit of Functional Proteomics, Metabolomics, and Network Analysis, 20138 Milan, Italy; sonia.eligini@cardiologicomonzino.it (S.E.); marco.munno@cardiologicomonzino.it (M.M.); 2 Department of Biological Chemistry, Institute of Life Sciences, The Hebrew University of Jerusalem, Jerusalem 91904, Israel; daphne.atlas@mail.huji.ac.il

**Keywords:** platelet, aggregation, N-acetylcysteine, N-acetylcysteine amide, thioredoxin-mimetic peptides, clotting time, TxB_2_, 12-HETE

## Abstract

In the present study, we tested the effect of small-molecular-weight redox molecules on collagen-induced platelet aggregation. We used N-acetylcysteine amide (AD4/NACA), the amide form of N-acetylcysteine (NAC), a thiol antioxidant with improved lipophilicity and bioavailability compared to NAC, and the thioredoxin-mimetic (TXM) peptides, TXM-CB3, TXM-CB13, and TXM-CB30. All compounds significantly inhibited platelet aggregation induced by collagen, with TXM-peptides and AD4 being more effective than NAC. The levels of TxB_2_ and 12-HETE, the main metabolites derived from the cyclooxygenase and lipoxygenase pathways following platelet activation, were significantly reduced in the presence of AD4, TXM peptides, or NAC, when tested at the highest concentration (0.6 mM). The effects of AD4, TXM-peptides, and NAC were also tested on the clotting time (CT) of whole blood. TXM-CB3 and TXM-CB30 showed the greatest increase in CT. Furthermore, two representative compounds, TXM-CB3 and NAC, showed an increase in the anti-oxidant free sulfhydryl groups of plasma detected via Ellman’s method, suggesting a contribution of plasma factors to the antiaggregating effects. Our results suggest that these small-molecular-weight redox peptides might become useful for the prevention and/or treatment of oxidative stress conditions associated with platelet activation.

## 1. Introduction

The antioxidant, N-acetylcysteine (NAC), a synthetic derivative of L-cysteine, and a precursor of glutathione (GSH) is known to protect against a variety of pathological conditions involving neurodegenerative disorders, cardiovascular diseases, inflammation, and carcinogenesis [1]. NAC is available in pharmaceutical formulations for oral, intravenous, and respiratory administration, and shows low toxicity with mild adverse effects such as gastrointestinal disorders, headache, erythema, hypotension, and bronchospasm [2,3].

Although it is typically safe and well tolerated even at high doses, the bioavailability of NAC is low (about 5%) [4,5] since, at a physiological pH, the carboxyl group is negatively charged, preventing membrane permeability [6].

In order to improve bioavailability, NAC has been amidated to N-acetylcysteine amide (AD4, also called NACA) [6]. AD4/NACA has shown enhanced antioxidant, antiapoptotic, and anti-inflammatory properties, compared to its parent compound. Moreover, AD4 can replenish intracellular GSH through the thiol–disulfide exchange with oxidized glutathione (GSSG), or by acting as a precursor to GSH [6,7,8,9,10].

Pathological conditions related to oxidative stress are often associated with the activation of stress-response proteins and antioxidant enzymes, including the thioredoxin reductase/thioredoxin (TrxR/Trx) system [11]. The TrxR/Trx system is one of the main cellular redox systems, which is highly conserved from yeast to mammals, and uses nicotinamide adenine dinucleotide phosphate (NADPH) as co-substrate. Through a thiol–disulfide exchange reaction, this system maintains the thiol–disulfide redox balance and protects cells from oxidative stress. In particular, the unique and highly conserved -CxxC- motif is responsible for the redox activity of thioredoxin. Tri-and tetra-peptides designed based on the CxxC motif were shown to mimic the redox activity of thioredoxin in vitro and in vivo [8,11,12,13,14,15,16,17,18,19]. These peptides, called thioredoxin-mimetic (TXM) peptides, include N- and C-terminal-blocked tri- and tetra-oligopeptides derived from the -CxxC- motif of thioredoxin.

TXM peptides scavenge free radicals, display anti-inflammatory activity, prevent NF-kB nuclear translocation, and inhibit mitogen-activated protein kinases. Additionally, the TXM-peptides exhibit a greater potency compared to NAC or AD4, both in vitro and in vivo [8,11,12,13,14,15,16,17,18,19].

Recently, we have shown that NAC inhibits platelet function, reducing aggregation, adhesion to collagen matrix, ROS generation, and intracellular calcium mobilization [20].

The aim of this study was to examine the effects of NAC-amide (AD4/NACA) and the TXM peptides, TXM-CB3, TXM-CB13, and TXM-CB30 on platelets by assessing their aggregability, activation, and the oxidative status of their environment.

## 2. Materials and Methods

### 2.1. Blood Collection and Platelet Preparation

The study was performed according to the Declaration of Helsinki and was approved by the local institutional Ethics Committee. Twenty male and female healthy subjects (aged 25–60 years) were enrolled. All the participants provided written informed consent at the time of enrollment and none of the participants had taken any anti-inflammatory drugs, nor any drugs known to affect platelet function, in the previous 10 days. Venous blood was drawn from the antecubital vein and collected in Vacutainer® tubes containing 0.129 mM sodium citrate. Healthy donors had a platelet count between 250–350 × 10^3^/µL. Platelet-rich plasma (PRP) was obtained via centrifugation at 100× *g* for 10 min without pause.

For washed platelet (WP) preparation, blood was collected in a Vacutainer^®^ tube containing ACD (trisodium citrate 22 g/L, citric acid 8.0 g/L, dextrose 24.5 g/L) as an anticoagulant and PRP was obtained via blood centrifugation at 130× *g* for 20 min. After the addition of 1 µM prostaglandin E_1_ and 10 mM ethylenediaminetetraacetic acid (EDTA) to PRP, the platelet suspension was centrifuged at 1000× *g* for 10 min, and the pellet was resuspended in Tyrode’s HEPES albumin free buffer. The platelet count was adjusted at 300,000/µL with the same buffer. The washed platelet preparation was kept for 30 min at room temperature before the aggregation.

### 2.2. Platelet Aggregation

AD4 (NAC-amide/NACA) and thioredoxin mimetic peptides (TXM) TXM-CB3, CB13, CB30, were custom synthesized by Novetide, Ltd., Haifa, Israel. NAC (Sigma-Aldrich S.r.l., Milan, Italy). All compounds (60 mM stock solution) were dissolved in ammonium formate buffer with a pH of 3.5 (vehicle).

Platelet aggregation was carried out in a PAP-8E BioData aggregometer (Bio/Data Corporation, Sentinel Diagnostic, Milan, Italy) at 37 °C, under constant stirring (1200 rpm). An amount of 250 µL of PRP was pre-incubated with vehicle, NAC, AD4, or TXM peptides (all 0.1–0.6 mM as indicated) for 30 min, at 37 °C under constant stirring. After the addition of collagen (0.5 µg/mL or 8 µg/mL for PRP and washed platelets, respectively), platelet aggregation was monitored for 6 min as the change in light transmittance. The results are expressed as the area under the curve (AUC).

### 2.3. Measurement of Thromboxane B_2_ and 12-hydroxyeicosatetraenoic Acid

At the end of the aggregation reaction, platelet aggregates were pelleted via centrifugation at 1500× *g*, and plasma was collected. The levels of thromboxane (Tx)B_2_, the stable metabolite of TxA_2_, and 12-HETE were measured using a liquid chromatography-tandem mass spectrometry (LC-MS/MS) method as previously described with some modifications [21]. The system used is ExcionLC coupled to a Qtrap 5500 mass spectrometer (Sciex, Milan, Italy) outfitted with Electrospray ionization (ESI) source and managed using Analyst software version 1.6.3. The Multiquant software, version 3.0.2, was used to process the acquired data. A chromatographic separation was performed using Xbridge BEH C18 column (2.1 × 30 mm, particle size 2.5 µm, Waters) at 40 °C. The mobile phase A was 50 mM ammonium acetate pH = 8/H_2_O/MeOH 4:93:3 *v*/*v*/*v*. The mobile phase B was 50 mM ammonium acetate pH = 8/acetonitrile (ACN)/methanol (MeOH) 4:93:3 *v*/*v*/*v*. The flow rate was 0.25 mL/min, and the total run time was 12 min. The gradient of the mobile phase is reported in Table 1.

Data acquisition was performed in the negative polarity mode, with the following source settings: curtain gas flow = 20; collisionally activated dissociation gas = medium; ion spray voltage = −3500; temperature = 400; gas 1 = 20, gas 2 = 30. The acquisition mode used was multiple reaction monitoring, with unit resolution for both quadrupole (Q1 and Q3). The transitions monitored, with their set parameters, are reported in Table 2.

TXB_2_ and 12-HETE standards were dissolved in MeOH (10 ng/µL) and serially diluted using H_2_O/MeOH/ACN 80:10:10 *v*/*v*/*v* (Solvent MIX) to prepare the calibration standard curve. The standard curve, ranging from 3.9 to 125.0 ng/mL for both the analytes, was spiked with the deuterated standards, TXB_2_-d_4_ and 12-HETE-d_8,_ both at 20.0 ng/mL. The concentrations of TXB_2_ and 12-HETE were calculated using the corresponding calibration curve based on the response ratio (peak area of unlabeled analyte/peak area of labeled internal standard).

### 2.4. Analysis of Platelet Function by Means of PFA-200 Analyzer

Platelet function was assessed using the platelet function analyzer, Innovance PFA-200 (Siemens Healthcare s.r.l., Milan, Italy). Citrate blood samples collected from healthy subjects were treated with vehicle, NAC, AD4, or TXM peptides (all at 0.6 mM) for 30 min, with agitation at 37 °C, and loaded onto the collagen/adenosine diphosphate (coll/ADP) or collagen/epinephrine (coll/epi) cartridges (Siemens Healthcare s.r.l., Milan, Italy). The incubation of whole blood with these agonists led to platelet activation and the formation of a primary clot. The time required for the occlusion of an aperture of 147 µm of diameter was recorded by the instrument, and is reported as closure time (CT) in seconds. The maximum CT measured by PFA-200 was 300 s. If CT was longer than 300 s, it was reported as 300 s.

### 2.5. Quantification of Sulfhydryl Groups

At the end of the aggregation process, samples were centrifuged, platelet aggregates were pelleted, and plasma was collected. Plasma and platelet sulfhydryl groups were detected using Ellman’s method modified by Riddles et al. [22,23]. Briefly, 50 µL of Ellman’s reagent (4 mg 5,5′-dithio-bis-(2-nitrobenzoic acid)/mL reaction buffer (0.1 M sodium phosphate, pH 8.0 containing 1 mM EDTA)) and 2.5 mL of reaction buffer were added to 250 µL of the sample. After 15 min of incubation at room temperature, absorbance was measured at 412 nm. Ellman’s reagent has a highly oxidizing disulfide bond, which was reduced by free sulfhydryl groups to form quantifiable, yellow-colored 2-nitro-5-thiobenzoic acid. The concentration of sulfhydryl groups in the sample was calculated using the molar extinction coefficient 14,150 M^−1^ cm^−1^.

### 2.6. Measurement of the Plasma Antioxidant Activity

The antioxidant activity was assessed in the plasma samples obtained before and after platelet aggregation, and subsequent removal of platelets and aggregates via centrifugation. The fluorometric assay was performed using the 2′,7′-dichlorodihydrofluorescin diacetate (DCFH-DA; Sigma-Aldrich S.r.l., Milan, Italy) as probe ad 2,2′-Azobis (2-amidinopropane) dihydrochloride (AAPH, Sigma-Aldrich S.r.l., Milan, Italy) as the radical generator. DCFH was prepared from DCFH-DA via basic hydrolysis: 500 µL of 1 mM DCFH-DA was mixed at 4 °C with 2 mL 0.01 M NaOH while protected from the light. After 20 min, the mixture was neutralized with 2 mL 0.01 M HCl and diluted with phosphate-buffered saline (PBS; 10 µM final concentration). An amount of 20 µL of sample was loaded in triplicate in a 96-well black plate (Corning Incorporated Costar, Euroclone S.p.A., Milan, Italy) and diluted with 50 µL PBS; 20 µL AAPH (10 mM final concentration) and 10 µL DCFH (10 µM final concentration) were added. Oxidation of DCFH to 2′,7′-dichlorofluorescin (DCF) was monitored at 37 °C, setting the excitation at λ 485 nm and emission at λ 535 nm.

### 2.7. Statistical Analysis

Data are expressed as mean ± SD. Differences between the groups were assessed using the Student’s *t*-test for single comparison or via the analysis of the variance for repeated measures (ANOVA) and Dunnett’s post hoc test. A *p*-value < 0.05 was considered significant.

## 3. Results

### 3.1. N-acetylcysteine Amide (AD4), Thioredoxin-Mimetics (TXM-Peptides), and N-acetylcysteine (NAC) Inhibit Platelet Aggregation

**Figure 1:**
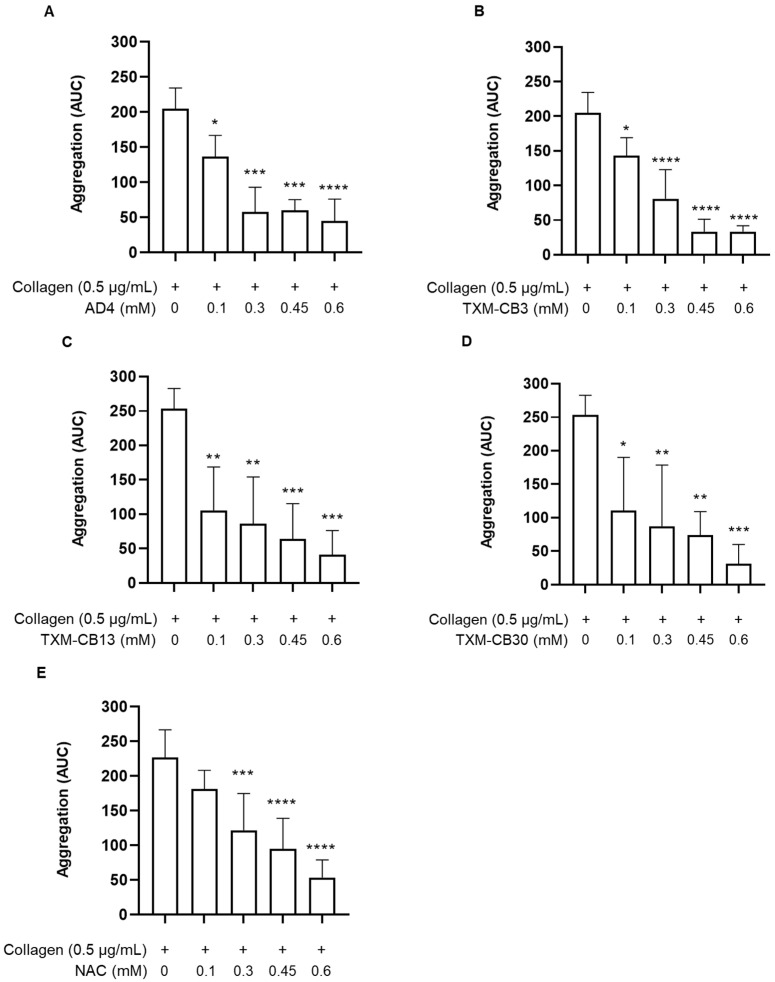
Inhibition of platelet aggregation by N-acetylcysteine amide (AD4), thioredoxin mimetics (TXM-CB3, TXM-CB13, TXM-CB30), and N-acetylcysteine (NAC). PRP was incubated with the vehicle or the different compounds for 30 min at 37 °C with constant stirring. Subsequently, platelets were stimulated with collagen (0.5 µg/mL). Concentration-dependent inhibition of platelet aggregation induced by collagen (0.5 µg/mL) after pretreatment with (**A**) AD4, *n* = 3; (**B**) TXM-CB3, *n* = 4; (**C**) TXM-CB13, *n* = 4; (**D**) TXM-CB30, *n* = 4; (**E**) NAC, *n* = 7. * *p* < 0.05, ** *p* < 0.01, *** *p* < 0.001, **** *p* < 0.0001 vs. collagen-stimulated PRP for ANOVA and Dunnett’s post hoc test.

PRP obtained from healthy subjects was preincubated for 30 min at 37 °C in the presence of the vehicle or with increasing concentrations of AD4, NAC, and the corresponding TXM-peptides, TXM-CB3 (AcCysProCysNH_2_), TXM-CB13 (AcCysMetLysNH_2_), and TXM-CB30 (AcDCysGlyDCysNH_2_). Subsequently, platelet aggregation was induced via the addition of collagen (0.5 µg/mL) and was monitored for 6 min. As shown, all the TXM-peptides and AD4 inhibited platelet aggregation in a concentration-dependent manner (Figure 1A–D), similar to the extent detected with NAC (Figure 1E). Inhibition of platelet aggregation by AD4 and the TXM-peptides was apparent already at 0.1 mM, compared to the lesser effect of NAC at this concentration, indicating their superior efficacy.

### 3.2. N-acetylcysteine Amide (AD4), Thioredoxin Mimetics (TXM Peptides), and N-acetylcysteine (NAC) Prevent the Generation of TXB_2_ and 12-HETE

**Figure 2:**
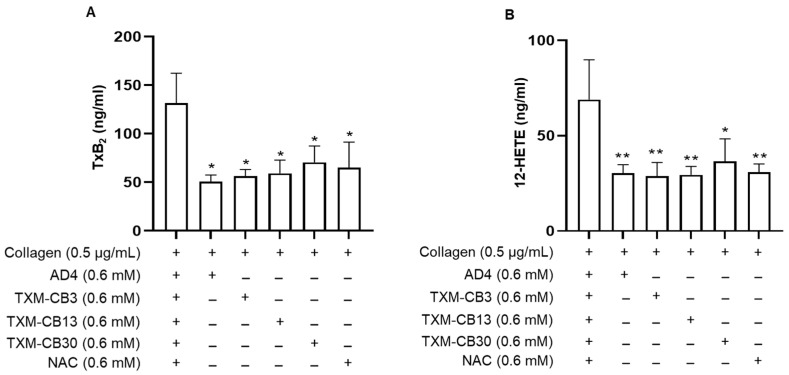
Inhibition of TxB_2_ and 12-HETE synthesis by N-acetylcysteine amide (AD4), thioredoxin-mimetics (TXM-CB3, TXM-CB13, TXM-CB30), and N-acetylcysteine (NAC). PRP was incubated with the vehicle or the different compounds for 30 min at 37 °C with constant stirring. Subsequently, platelets were stimulated with collagen (0.5 µg/mL). At the end of aggregation, platelet aggregates were pelleted and the levels of (**A**) TxB_2_ and (**B**) 12-HETE were simultaneously measured. *n* = 3. * *p* < 0.05, ** *p* < 0.01, vs. collagen-stimulated PRP for ANOVA and Dunnett’s post hoc test.

Platelet activation triggers the arachidonic acid cascade, which results in generating TxA_2_, the main metabolite derived from the cyclooxygenase pathway, and 12-HETE, the metabolite derived from the lipoxygenase pathway. The levels of these metabolites were determined via quantitative mass spectrometry analysis. The analysis was performed on the supernatant obtained after collagen-induced platelet aggregation in the absence or in the presence of AD4, TXM-peptides, or NAC at 0.6 mM, the highest concentration. As shown in Figure 2, both AD4, NAC and the TXM-peptides significantly reduced the synthesis of both TxB_2_ and 12-HETE.

### 3.3. N-acetylcysteine Amide (AD4), Thioredoxin Mimetics and NAC Influence PFA-200 Closure Time

**Figure 3:**
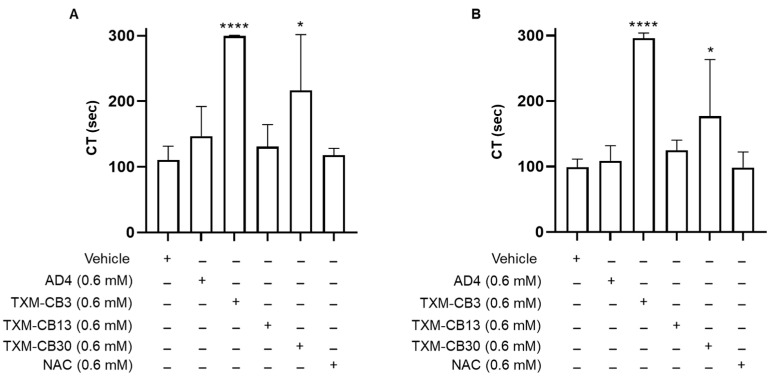
N-acetylcysteine amide (AD4), thioredoxin-mimetics (TXM-CB3, TXM-CB13, TXM-CB30), and N-acetylcysteine (NAC) prolonged the closure time (CT) in PFA-200 analysis. Whole blood was incubated for 30 min at 37 °C and perfused in a (**A**) coll/epi or (**B**) coll/ADP cartrige of the PFA-200 system, and the CT was recorded. *n* = 4. * *p* < 0.05, **** *p* < 0.0001, vs. vehicle-incubated blood for ANOVA and Dunnett’s post hoc test.

The PFA-200 system simulates a damaged blood vessel by aspirating blood through disposable test cartridges coated with collagen and either ADP (50 μg) or epinephrine (10 μg). Whole blood (800 μL) flows through a capillary and a microscopic aperture (147 μm) cut into the coated membrane under constant high shear rates (5000 to 6000/s). As blood contacts the membrane, platelets adhere, aggregate, and form a plug that occludes the aperture, causing blood flow to cease. The time required to occlude the aperture is automatically reported as CT. Measurements are terminated after 300 s. The effect of AD4, NAC, and TXM-peptides on CT of whole blood was measured in the PFA-200 system. AD4, TXM-CB3, TXM-CB13, and TXM-CB30, were tested at the highest concentration (0.6 mM). AD4 and TXM-CB13 showed a slight increase in CT in the coll/epi test (147 ± 45 s, 131 ± 34 s, respectively), while TXM-CB30 showed 216 ± 85 s, compared to vehicle-treated blood (111 ± 21 s). TXM-CB3 showed the greatest increase in CT (300 s), and NAC did not affect CT (Figure 3A).

Similarly, in the coll/ADP test, TXM-CB3, and TXM-CB30 prolonged the CT (~300 s and around 200 s, respectively) (Figure 3B).

### 3.4. N-acetylcysteine Amide (AD4), TXM-CB3 and NAC Do Not Inhibit Aggregation of Washed Platelet

**Figure 4:**
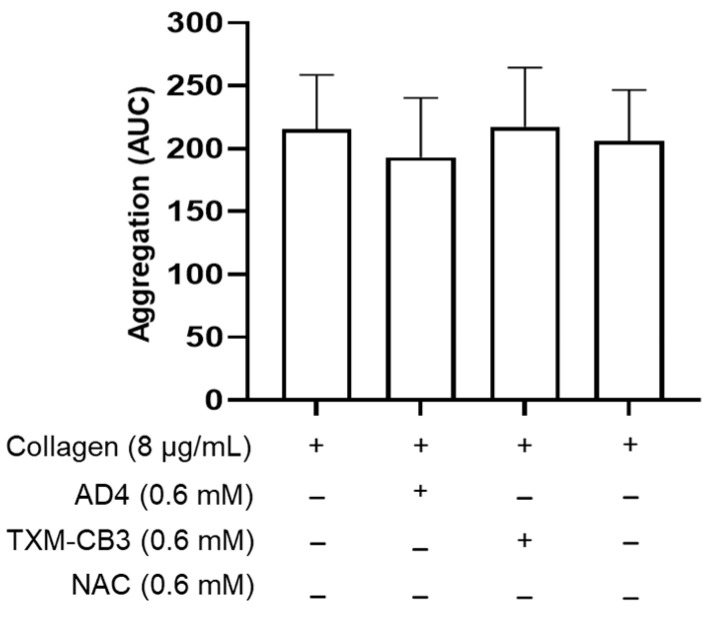
N-acetylcysteine amide (AD4), TXM-CB3, and NAC did not inhibit aggregation of washed platelets. Washed platelets were preincubated with the vehicle or AD4, or TXM-CB3, or NAC for 30 min at 37 °C with constant stirring, and subsequently stimulated with collagen (8 µg/mL). Aggregation was monitored for 6 min. *n* = 7.

In contrast to PRP, preincubation of washed platelets with AD4 or TXM-CB3 as representative of the TXM-peptides, did not inhibit collagen (8 µg/mL)-induced platelet aggregation (Figure 4). Similarly, the inhibitory activity of NAC on PRP aggregation was completely lost in washed platelet (Figure 4), indicating no direct effects on platelets in the absence of plasma components.

### 3.5. Thioredoxin-Mimetic TXM-CB3 and NAC Increase the Free Sulfhydryl Groups in Plasma but Not in Platelets

**Figure 5:**
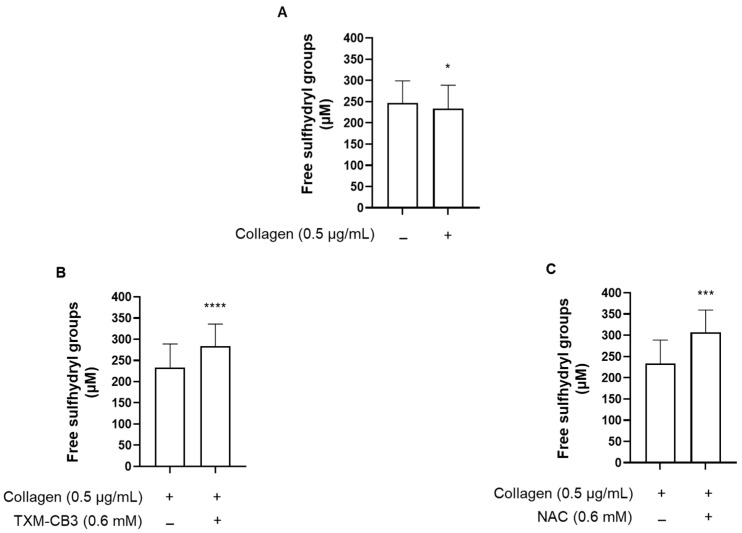
Quantitation of sulfhydryl groups. PRP was preincubated with the (**A**) vehicle, (**B**) thioredoxin mimetics (TXM-CB3), or (**C**) N-acetylcysteine (NAC), for 30 min at 37 °C with constant stirring. Platelets were stimulated with collagen (0.5 µg/mL). After 6 min, plasma was separated via centrifugation and free sulfhydryl groups were quantified using Ellman’s reagent. *n* = 5. * *p* < 0.05; *** *p* < 0.001; **** *p* < 0.0001 vs. collagen-stimulated PRP.

We then assessed whether the antiaggregant effects are dependent on the regeneration of free sulfhydryl groups. We measured free -SH groups in the plasma environment surrounding the platelets before and after aggregation, using Ellman’s reagent. Compared to basal condition, collagen-induced aggregation further decreased the levels of free sulfhydryl groups in plasma proteins (Figure 5A). Preincubation with TXM-CB3 or NAC, significantly increased the level of free sulfhydryl groups, which were detected in the plasma after PRP collagen stimulation, and subsequent to removal of platelet aggregates (Figure 5B,C).

Collagen stimulation of PRP induced a modest non-significant decrease in free sulfhydryl groups in platelets (36.53 ± 8.09 µM under basal condition, and 25.72 ± 9.81 µM after collagen-induced aggregation; *n* = 5). No change was observed in the levels of free sulfhydryl groups following preincubation of PRP with TXM-CB3 or NAC, compared to collagen-stimulated PRP (31.74 ± 4.12 µM and 29.58 ± 5.16 µM for TXM-CB3 and NAC, respectively vs. 25.72 ± 9.81 µM for collagen-stimulated PRP; *n* = 5). These results support no direct effect of these compounds on sulfhydryl groups in PRP.

### 3.6. Thioredoxin-Mimetic TXM-CB3 and NAC Increase the Total Plasma Antioxidant Activity

**Figure 6:**
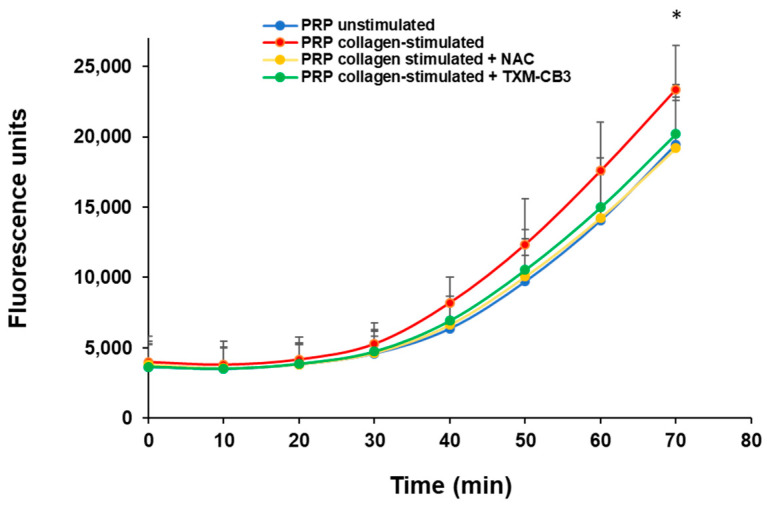
Total antioxidant activity. PRP was preincubated with vehicle, TXM-CB3, or NAC for 30 min at 37 °C with constant stirring. Platelets were stimulated with 0.5 µg/mL collagen. After 6 min plasma was separated via centrifugation and antioxidant activity was detected via DCF fluorescence. Data are expressed as fluorescence units of DCF. *n* = 5. * *p* < 0.05 vs. unstimulated PRP (70 min).

As regeneration of free thiols is one of the main protective mechanisms against oxidative stress, which ultimately leads to excessive platelet activation, we evaluated the antioxidant activity within the plasma samples before and after collagen-induced platelet aggregation. After the addition of AAPH as a radical generator, a lag time was observed and the propagation phase started after ~30 min (Figure 6). After 70 min of incubation, a marked increase in DCFH oxidation was detected in collagen-treated samples. In contrast, preincubation of PRP with TXM-CB3, or NAC completely prevented DCFH oxidation and the total antioxidant activity measured in these samples was similar to that detected in unstimulated PRP samples (Figure 6).

## 4. Discussion

Inhibition of platelet aggregation by antioxidant/anti-inflammatory agents may prove to be efficacious in the treatment of cardiovascular diseases. Here, we show that the amino-acid-based compounds, NAC-amide (AD4/NACA), and the thioredoxin mimetic peptides TXM-CB3, TXM-CB13, and TXM-CB30 inhibit collagen-induced platelet aggregation.

Compared to NAC, all these thiol-containing peptides exhibited a significantly higher antiaggregant activity. Indeed, in previous studies the TXM peptides have been shown to display a higher redox potency over NAC and AD4. This higher potency is attributed mainly to having two Cys residues, and to the cluster effect associated with the TXM peptides [8].

Platelet activation involves the mobilization of arachidonic acid from phospholipids of the plasma membrane, which is rapidly converted to Tx and prostaglandins from cyclooxygenase and hydroxyeicosatetraenoic acid from lipoxygenase enzymes. In platelets, the major metabolites derived from these two metabolic pathways are TxA_2_ and 12-HETE synthesized from cyclooxygenase and lipoxygenase, respectively [24]. While it is known that TxA_2_ is a potent mediator of platelet activation and contributes to sustaining platelet aggregation through the activation of the TxA_2_ receptor on platelets, the role of 12-HETE in platelet function remains less clear, but has been suggested to contribute to both platelet adhesion and aggregation [25,26]. The mechanism(s) by which all these compounds inhibit the synthesis of the arachidonic acid metabolites is unknown; however, it has been shown that NAC reduced the mobilization of arachidonic acid in macrophages with concomitant inhibition of the release of the inflammatory lipid mediators, prostaglandin E_2_ and TxB_2_ [27]. The present results demonstrate a marked inhibition of both TxB_2_, a stable metabolic product of TxA_2_, and 12-HETE after PRP treatment with the TXM peptides, NAC, and AD4.

The PFA-200 system was designed as a global assay for platelet-dependent primary hemostasis; however, besides the detection of platelet dysfunction, it may be also be used for monitoring anti-platelet therapy by assessing the contribution of systemic inflammation onto platelet function [28,29]. Through this ex vivo test, we found that all compounds prolonged CT in the PFA-200 system. Among them, the most marked effect was exerted by TXM-CB3 and TXM-CB30 both in coll/ADP and coll/epi tests, suggesting that they interfere with the binding of epinephrine and ADP to the corresponding platelet receptors.

Establishing the potency and efficacy of different redox molecules requires the determination of dissociation constants using extensive concentration-dependency studies. The most effective increase in the CT of whole blood by the tri-peptides TXM-CB3 and TXM-CB30, as shown in the present study, suggests an optimal activity associated with peptide length (a tri-peptide vs. a tetra-peptide (TXM-CB13). However, more studies are required to establish such a correlation as well as for showing a difference between the all-L-TXM-CB3 and all-D TXM-CB30.

It is known that the effect of ADP on platelets is mediated by three purinergic receptors, designated P2Y1, P2Y12, and P2X1 [30]. While the P2X1 receptor is an ion channel that, upon activation, induces calcium influx and does not lead to aggregation by itself, the activation of the other two purinergic receptors results in platelet aggregation. Additionally, the activation of the P2Y1 receptor leads to changes in the shape of platelets, TxA_2_ generation, adhesion to fibrinogen, and to aggregation. Similarly, P2Y12 activation sustains the aggregation and potentiates the platelet activation induced by other physiological agonists including collagen, von Willebrand, and TxA_2_. The importance of this receptor is linked to the use of antithrombotic drugs such as clopidogrel, ticlopidine, and prasugrel, which, through blocking the P2Y12 receptor, inhibit platelet activation and reduce the risk of heart attack and stroke [30]. In contrast to the P2Y1 receptor, which has two disulfide bridges in the extracellular domain, the P2Y12 receptor shows two free cysteines (Cys17 and Cys270) essential for its activity. Therefore, it has been proposed that clopidogrel, as well as other thiol reagents, inactivate the P2Y12 receptor through the formation of disulfide bridges [30].

With regard to the effect on coll/epi, it is known that the catecholamines, epinephrine and norepinephrine, induce platelet aggregation through binding to the α-adrenergic receptor and inhibition of adenylate cyclase activity [31]. It has been demonstrated that stimulation of downstream targets of the α-adrenergic receptor requires an oxidative modification of thiols [32]. This has led to the supposition that reducing the activity of the compounds is likely to affect the mediator(s) involved in platelet activation.

The lack of an effect on the CT by NAC, as shown in the present study, can be explained by the use of a lower concentration compared to previous studies (>2 mM). The authors suggested a reduction of disulfide bonds of von Willebrand factor, a multimeric plasma protein that mediates the adhesion and aggregation of platelets at the site of a vascular injury [33].

In contrast to what was detected in PRP, no inhibitory effect of the thiol compounds was observed on collagen-induced aggregation in washed platelets suspended in the albumin-free Tyrode’s HEPES buffer. These results have led us to hypothesize that the factors present in the plasma are likely to be affected. Previously, we have demonstrated that NAC inhibits platelet activation through the regeneration of the antioxidant form of plasma albumin [20,34]. Further studies are therefore required to understand the mechanism(s), including the role of albumin, by which the thiol-based reagents, AD4, and TXM-peptides modulate platelet activation.

It is known that reactive oxygen species (ROS) affect platelet function not only by causing aggregation, but also by modulating intracellular signaling pathways [35,36]. Furthermore, in addition to being targeted by ROS, platelet activation itself induces ROS formation, promoting platelet aggregation [37,38,39].

We have previously demonstrated that collagen-induced stimulation of platelets resulted in a marked increase in ROS generation [20]. Here, we have shown that the protective effect of the thiol reagents is mainly due to an increase in free sulfhydryl groups of proteins. This novel finding of an increase in free sulfhydryl groups by NAC and TXM-CB3 indicates regeneration in the plasma compartment after collagen-induced platelet aggregation. The sulfhydryl groups of proteins are mainly responsible for their antioxidant properties and have been demonstrated to contribute ~53% to the total antioxidant capacity of serum in healthy subjects [40]. Under our experimental conditions, platelet stimulation with collagen led to a significant reduction of the free sulfhydryl group measured in the plasma after the removal of platelet aggregates. Preincubation of PRP with TXM-CB3 appeared to prevent the reduction in free sulfhydryl groups, suggesting that by reducing oxidized proteins, the TXM peptide was able to maintain the plasma redox status and protect platelets from oxidative stress.

The plasma compartment represents an important defense system against ROS that are continuously generated in the body, including the redox enzymes, e.g., superoxide dismutase, glutathione peroxidases and catalase, protein thiols, and uric acid, and exogenous antioxidants such as ascorbic acid, vitamin E, tocopherols, and carotenoids [41,42]. We measured the total antioxidant capacity of plasma obtained after the activation of platelets with collagen and the removal of aggregates, using the radical generator, AAPH, and the hydrophilic oxidable substrate, DCFH. After a short lag time, a marked increase in the generation of DCF, the oxidized form of DCFH, was observed in the collagen-treated samples compared to unstimulated samples, and was completely prevented in the presence of TXM-CB3 or NAC. Although the antioxidant activity of the TXM peptides has been shown to be significantly more potent than NAC [19], both TXM-CB3 and NAC showed similar antioxidant activity. Further concentration-dependent studies are required to evaluate this discrepancy.

## 5. Conclusions

This study has shown, for the first time, the antiaggregating activity of N-acetylcysteine amide (AD4), the amide form of N-acetylcysteine (NAC), a thiol antioxidant with improved lipophilicity and bioavailability compared with NAC, and the thioredoxin mimetic (TXM) peptides, TXM-CB3, TXM-CB13, and TXM-CB30. The findings show that they act by inhibiting the generation of arachidonic acid metabolites following platelet activation through interfering with whole blood clotting, and regenerating the plasma antioxidant-free sulfhydryl groups. Overall, these data suggest that these small-molecular-weight TXM-redox peptides might become useful tools for the preventing and/or treatment of oxidative stress conditions associated with platelet activation.

## Figures and Tables

**Table 1:** Mobile phase gradient for the determination of TxB_2_ and 12-HETE.

Time (min)	% Mobile Phase A 50 mM Ammonium Acetate pH = 8/H_2_O/MeOH 4:93:3 *v*/*v*/*v*	% Mobile Phase B 50 mM Ammonium Acetate pH = 8/ACN/MeOH 4:93:3 *v*/*v*/*v*
0.0	85	15
0.2	85	15
2.0	65	35
2.5	15	85
6.0	15	85
6.5	85	15
12.0	85	15

**Table 2:** Mass transitions.

Q1 (*m*/*z*)	Q3 (*m*/*z*)	Name
369.2	169.1	TXB_2_ Quant
369.2	195.2	TXB_2_ Qual
373.2	173.0	TXB_2_-d4 Quant
373.2	199.2	TXB_2_-d4 Qual
319.1	179.0	12HETE Quant
319.1	301.2	12HETE Qual
327.2	184.2	12HETE-d8 Quant
327.2	309.3	12HETE-d8 Qual

## Data Availability

Data collected in the study will be made available using the data repository, Zenodo (https://zenodo.org; accessed on 17 July 2023), with restricted access upon request to direzione.scientifica@ccfm.it. Any remaining information can be obtained from the corresponding author upon reasonable request.
